# Comparative bioinformatics analysis of the Wnt pathway in breast cancer: Selection of novel biomarker panels associated with ER status

**DOI:** 10.1515/biol-2025-1173

**Published:** 2025-10-08

**Authors:** Klaudia Waszczykowska, Damian Kołat, Żaneta Kałuzińska-Kołat, Elżbieta Płuciennik

**Affiliations:** Department of Functional Genomics, Medical University of Lodz, Zeligowskiego 7/9, 90-752, Lodz, Poland; Department of Biomedicine and Experimental Surgery, Medical University of Lodz, Narutowicza 60, 90-136, Lodz, Poland

**Keywords:** breast cancer, Wnt signaling, carcinogenesis, biomarker signatures, cancer biomarkers

## Abstract

Breast cancer (BC) is a major global health concern, ranking among the most common neoplasms and representing one of the leading causes of cancer-related deaths worldwide. Early recognition and classification of BC subtypes are crucial for improving patient outcomes. Therefore, identifying novel biomarkers with diagnostic and prognostic significance is of great importance. The Wnt signaling pathway plays a significant role in BC by influencing various cell cycle regulation processes and stem cell renewal. This study aims to identify novel Wnt-associated biomarker panels for BC patients, composed of multiple molecular factors. A series of bioinformatical analyses have been employed, including weighted gene co-expression network analysis, differential expression analysis, Kaplan–Meier survival analysis, logistic regression model evaluation, and receiver operating characteristic construction. Thus, this study revealed potential diagnostic and prognostic signatures based on comprehensive analyses of BC patient data sourced from The Cancer Genome Atlas database. Consequently, four gene signatures were constructed: two differentiate ER+ from ER-BC: *TTC8, SLC5A7,* and *PLCH1* for overall survival (OS); *ZNF695, SLC7A5,* and *PLCH1* for disease free survival (DFS), while the other two effectively distinguish tumor from normal samples: *SPC25, ANLN, KPNA2, SLC7A5* for OS; *SPC25, KIF20A, SKA3, DTL, CDCA3, ANLN, TTK, RAD54L, MYBL2, ZNF695,* and *SLC7A5* for DFS.

## Graphical abstract

**Figure:**
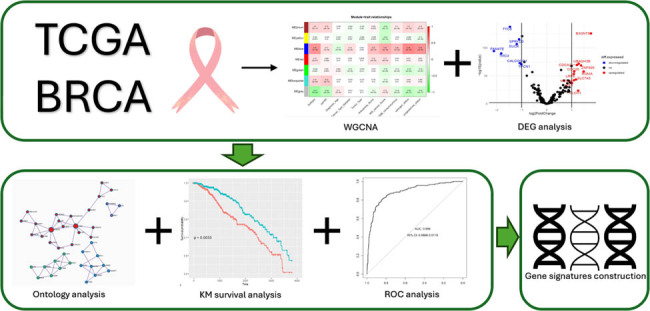

## 1 Introduction

Breast cancer (BC) is one of the most prevalent neoplasms across the world, being one of the leading causes of cancer-related deaths worldwide. In 2020, approximately 2.3 million cases were diagnosed, whereas 685,000 cases were fatal. BC is estimated to affect 4.4 million individuals by the year 2070 [1,2,3]. Molecular characteristics of BC include four intrinsic subtypes based on the status of estrogen receptor (ER), progesterone receptor (PR), and human epidermal growth factor receptor 2 (HER2/neu), namely luminal A, luminal B, HER2-enriched, and TNBC/basal-like (triple negative BC). Each subtype entails a different treatment approach, which is additionally modified by the unique molecular profile of each patient [4,5].

Currently, the major types of BC management include surgery, radiation therapy, chemotherapy, endocrine therapy, and targeted therapy. Each intervention serves a distinct purpose, and its selection depends on several factors, such as the type and stage of the malignancy, the individual’s general condition, and their preferences [6]. Apart from the selection of treatment, early recognition and characterization of BC type are key to the patient’s survival; thus, it is crucial to define novel biomarkers that could be of great diagnostic and prognostic importance [7,8,9]. Given the substantial molecular heterogeneity in the background of BC among individual patients, it is of great importance to establish personalized therapeutic interventions tailored to improve the chances of survival for each patient. An emphasized focus on advancing biomarker development holds the potential to develop innovative therapeutic strategies [10].

A signaling pathway that is often implicated in carcinogenesis, including BC, is the Wnt pathway. It influences embryonic development, being associated with various cell cycle regulation processes and stem cell renewal [11,12]. Its dysregulation in BC influences proliferation, metastasis, immune microenvironment regulation, and therapeutic resistance [13,14]. This study aimed to identify Wnt-associated molecular signatures as potential therapeutic targets, evaluated through bioinformatic analyses and literature review.

## 2 Materials and methods

### 2.1 Data acquisition and input gene selection

The data for this study were obtained from The Cancer Genome Atlas (TCGA) repository for BC patients (Breast Invasive Carcinoma (TCGA, PanCancer Atlas) obtained via the cBioPortal database (https://www.cbioportal.org/datasets), which included 1,082 BC patients and 114 matched normal samples. The genes that underwent further analysis were selected using the gene transcription regulation database (https://gtrd.biouml.org/) that consisted of 2,573 Wnt downstream effectors.

### 2.2 Weighted gene co-expression network analysis (WGCNA) and functional annotation

Pearson’s correlation between input genes was computed and progressed employing adjacency matrix transformation with the B-power = 5 and the scale-free topology fitting index (*R*_2_) > 0.9, following the standard guidelines for WGCNA [15]. Based on the connection between gene pairs, a topological overlap matrix was constructed to prepare hierarchical clustering using the hclust() function with the “average” method of agglomeration. The identification of modules was performed using the cutreeDynamic() function (minimum size of the module = 40, deep split level = 2). The obtained modules were further correlated with specific clinical traits of the BC patients. Furthermore, a logarithmically transformed *p*-value from linear regression facilitated the computation of the correlation’s significance between gene expression and specific clinical attributes. Finally, the collective importance of modules was calculated by averaging the significance of individual genes within a cluster of genes that are interconnected within the designated module. Disparities in expression patterns among distinct modules were graphically represented by employing the gplots package through the generation of heatmaps (via the heatmap.2() function), utilizing Pearson’s distance metric for arranging rows, and adopting the “complete” method of agglomeration. The PANTHER database (https://www.pantherdb.org/) was used to functionally annotate the selected modules of genes. The blue module, encompassing 183 genes, underwent subsequent analysis specifically in the context of BC patients.

### 2.3 Metascape protein–protein interaction enrichment analysis

Using Metascape (https://metascape.org), analysis and interpretation of blue module gene OMIC data were performed. The core outcomes focus on enrichment analysis, where genes found in the blue module of WGCNA were compared against multiple gene sets associated with various biological processes, protein functions, pathways, and other features. The input gene list (183) was extracted as the blue module from WGCNA (Table S1).

### 2.4 Differential expression analysis (DEA)

The differentially expressed genes (DEGs) were identified using Bioconductor’s package edgeR in two separate comparisons: ER-positive versus ER-negative patients, as well as normal versus tumor samples. Intergroup comparisons were established using the makeContrasts() function, which was preceded by using glmFit() and glmLRT() functions. The topTags() function identified and tabulated the most pronounced DEGs between the groups. A more restrictive cutoff of log2FC ≥3.5 was applied to identify the most robust and biologically significant differentially expressed genes; the parameters of log2FC ≥3.5 and a significance threshold of *p* < 0.01 were employed for DEG identification. The visual representation of these genes was achieved through volcano plots, employing the ggrepel package and its geom_text_repel() function. Two volcano plots showed gene expression distributions based on the ER status (“ER+” vs “ER−”) and sample type (“tumor” vs “normal” specimens).

### 2.5 Survival analysis

Genes with the most significant downregulation or overexpression were analyzed for disease-free survival (DFS) and overall survival (OS) using R libraries (survminer, survival, tidyverse) for both comparisons: “normal” vs “tumor” and “ER+” vs “ER−.” Next, genes exhibiting statistically significant individual survival analyses (*p*-value <0.05) were combined into distinct multi-gene signatures. These signatures underwent further analysis using R libraries (survminer, survival, tidyverse). Median gene expression was selected as a cutoff value for survival analyses to provide more balanced group comparisons and avoid small subgroups. The generation of survival plots was accomplished through the application of the ggplot2 package.

### 2.6 Logistic regression model of selected genes and receiver operating characteristic (ROC) evaluation with prediction assessment and validation of the model

To assess the overall diagnostic performance of the selected gene signatures, a linear regression model was built, and its potential was evaluated with ROC curves. To construct a logistic regression model, the cohort of BC patients was partitioned into two distinct training and testing subgroups, comprising 70 and 30% of the patients, respectively. The former subgroup was utilized for model development, whereas the latter group served for control purposes. The potential predictive capability of the novel marker signatures was examined using the ROC curve generated using the pROC R package. This analysis was carried out considering the presence of ER within cancer samples, as well as the distinction between tumor and normal tissue. The model was established using the glm function in *R*.

## 3 Results

### 3.1 Weighted gene correlation network analysis revealed genes most closely correlated with the ER status of BC patients

**Figure 1:**
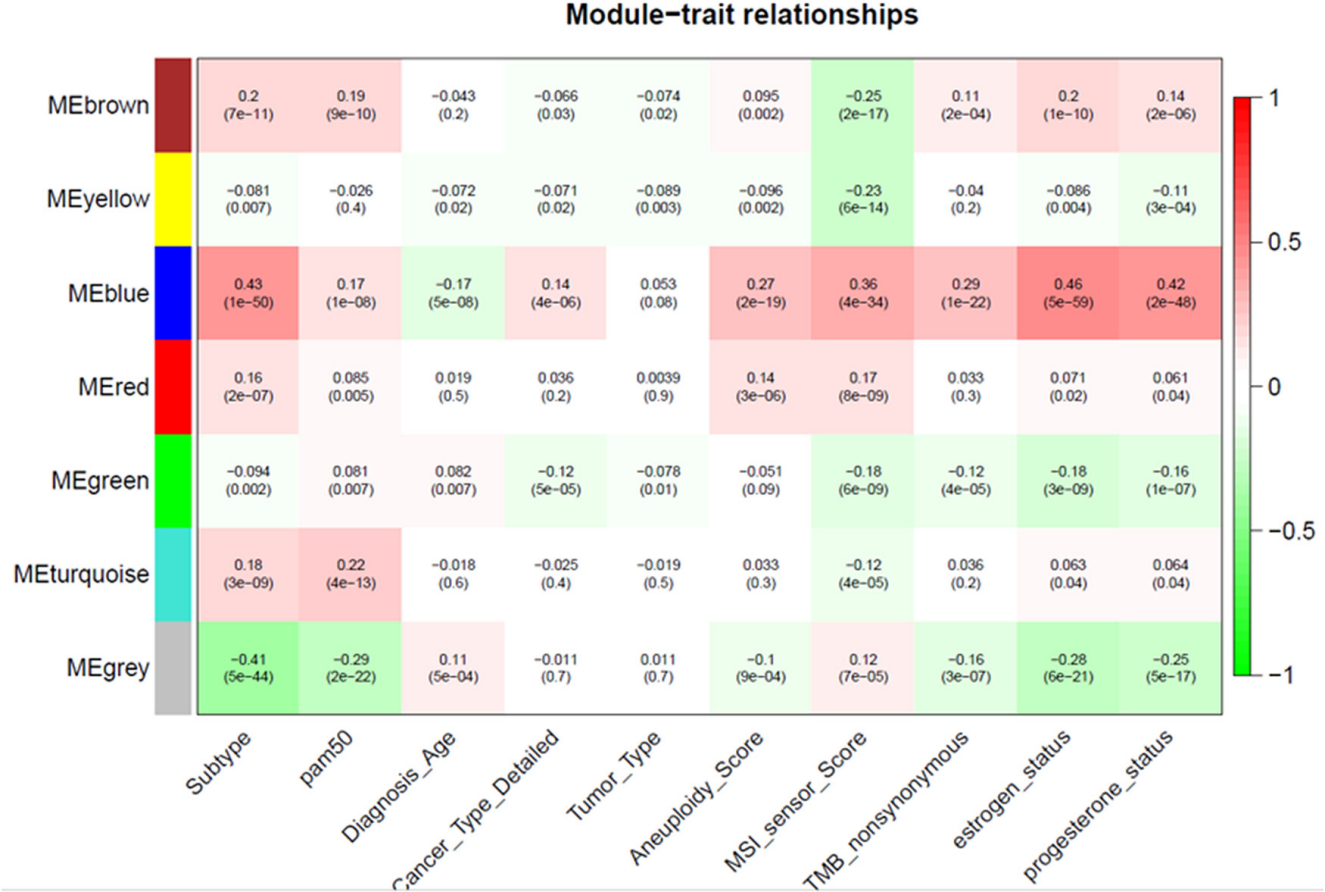
The weighted gene correlation network analysis heatmap showing correlation between genes grouped into modules of similar expression patterns and particular clinical traits of BC patients. Negative correlation is represented by green color, while positive correlation is marked with red. The greatest correlation was found for genes included in the blue module and ER status (0.46; *p* < 0.05).

A statistically significant correlation of *R* = 0.46 was noted between the genes included in the blue module and the status of ER (Figure 1). This particular module comprised 183 genes, which subsequently underwent further analysis to evaluate their prognostic potential and identify gene functionality, alongside their potential implications for the survival of patients diagnosed with BC. Detailed information on genes included in the blue module is provided in Table S1.

### 3.2 Enrichment analysis with Metascape revealed functional associations of blue module genes

**Figure 2:**
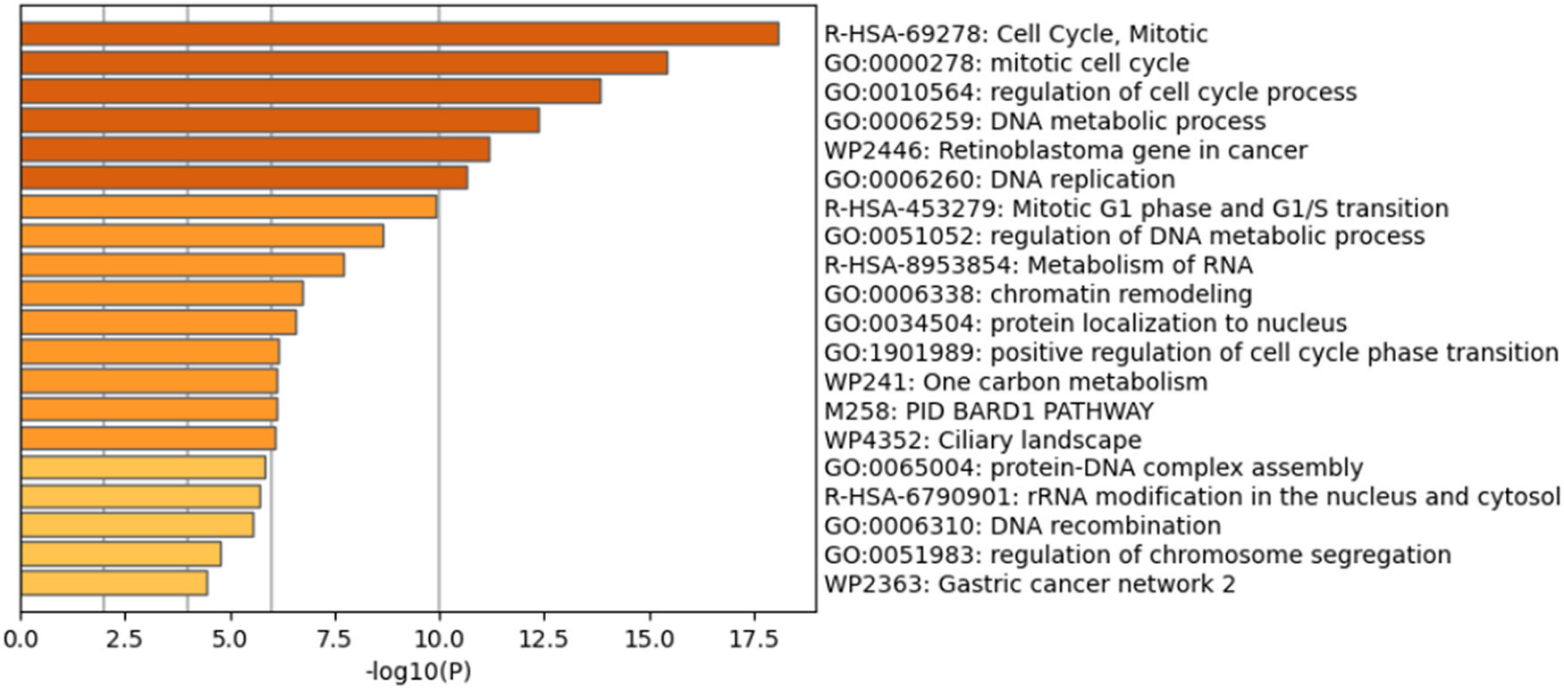
Bar graph showing the enriched processes across the input gene lists, with darker colors indicating higher statistical significance of the displayed terms. Pathway and process enrichment analysis was performed for each gene list using the following ontology sources: KEGG pathway, GO biological processes (GO), Reactome gene sets (R-HSA), canonical pathways, CORUM, Wiki pathways (WP), and PANTHER pathway. The entire genome served as the enrichment background.

In the subsequent phase of analysis, gene sets that were significantly enriched were analyzed to derive potential biological insights relevant to the study (Figure 2).

**Figure 3:**
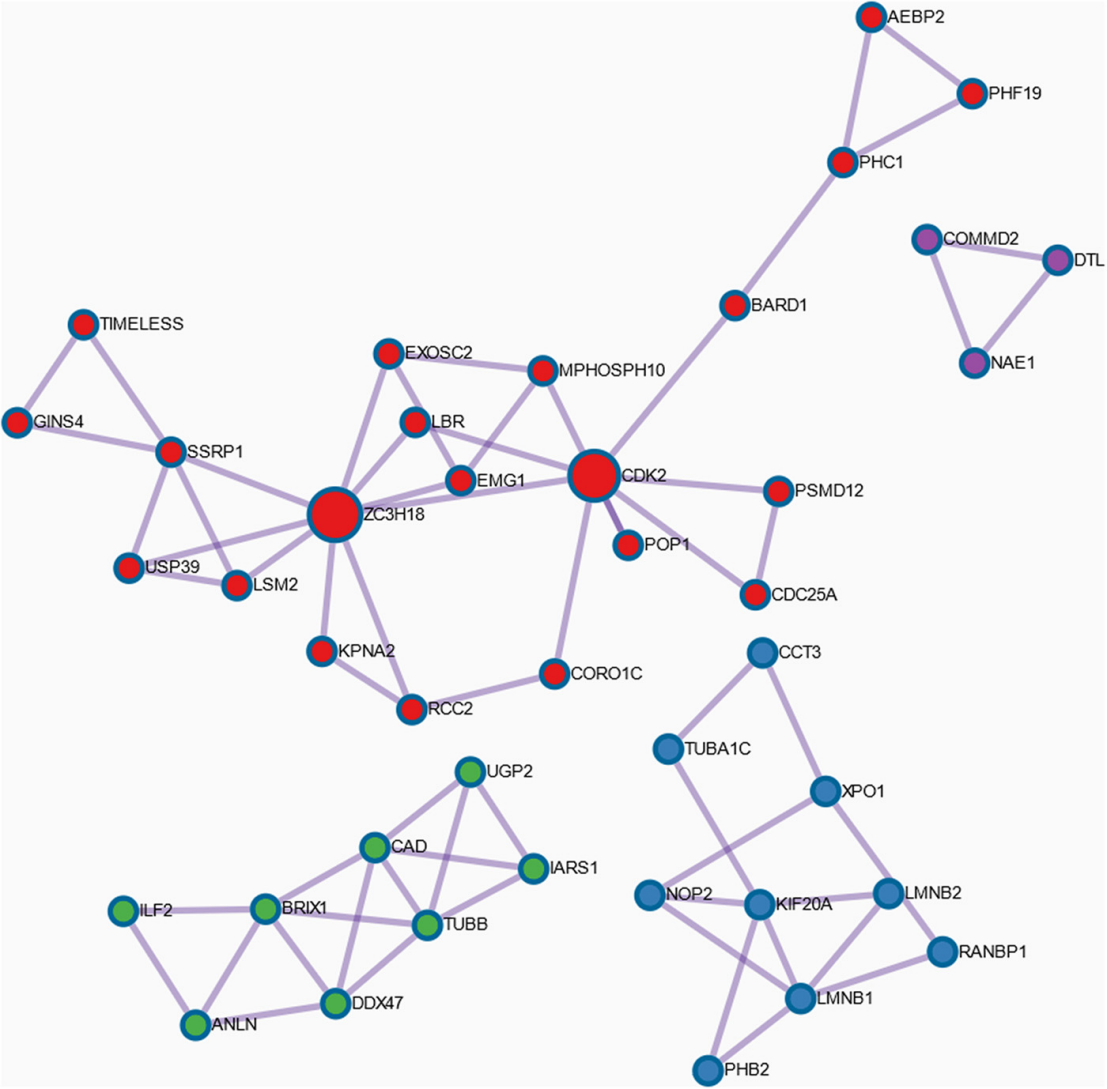
The four major networks for the provided set of ER-associated Wnt genes.

Metascape enrichment analysis revealed that genes within the blue module are significantly linked to cell cycle processes, particularly the mitotic cycle (16%; *p* < 0.05). Additionally, these genes showed a strong association with DNA metabolic processes (14.21%; *p* < 0.05). Also, 11 genes (6.01%; *p* < 0.05) were identified as connected to the retinoblastoma pathway in cancer. Processes that are less frequently linked to this gene module, but still hold significance, include DNA replication, RNA metabolism, chromatin remodeling, and protein–DNA complex assembly. Detailed information on these specific processes can be found in Table S2. In addition, several groups of genes were organized into clusters based on the protein–protein interactions identified (Figure 3).

Four major interaction networks were identified during this step: *AEBP2, BARD1, CDK2, CDC25A, CORO1C, EMG1, EXOSC2, GINS4, KPNA2, LBR, LSM2, MPHOSPH10, PHC1, PHF19, POP1, PSMD12, RCC2, SSRP1, TIMELESS, USP39*, and *ZC3H18* as the red network; *CCT3, KIF20A, LMNB1, LMNB2, NOP2, PHB2, RANBP1, TUBA1C*, and *XPO1* as the blue network*; ANLN, BRIX1, CAD, DDX47, IARS1, ILF2, TUBB*, and *UGP2* as the green network; and *COMMD2, DTL*, and *NAE1* as the purple network. Each network was found to be linked with distinct biological processes, which included DNA replication, cell cycle progression, and RNA metabolism (red nodes); protein localization to the nucleus, apoptosis, and cell cycle M phase (blue nodes); the assembly of non-membrane-bound organelles (green nodes); and the neddylation process (purple nodes). A detailed description of processes and *p*-value scores is provided in Table S3.

### 3.3 DEGs were identified by comparing ER-positive and negative BC patients, as well as tumor and normal breast tissue

**Figure 4:**
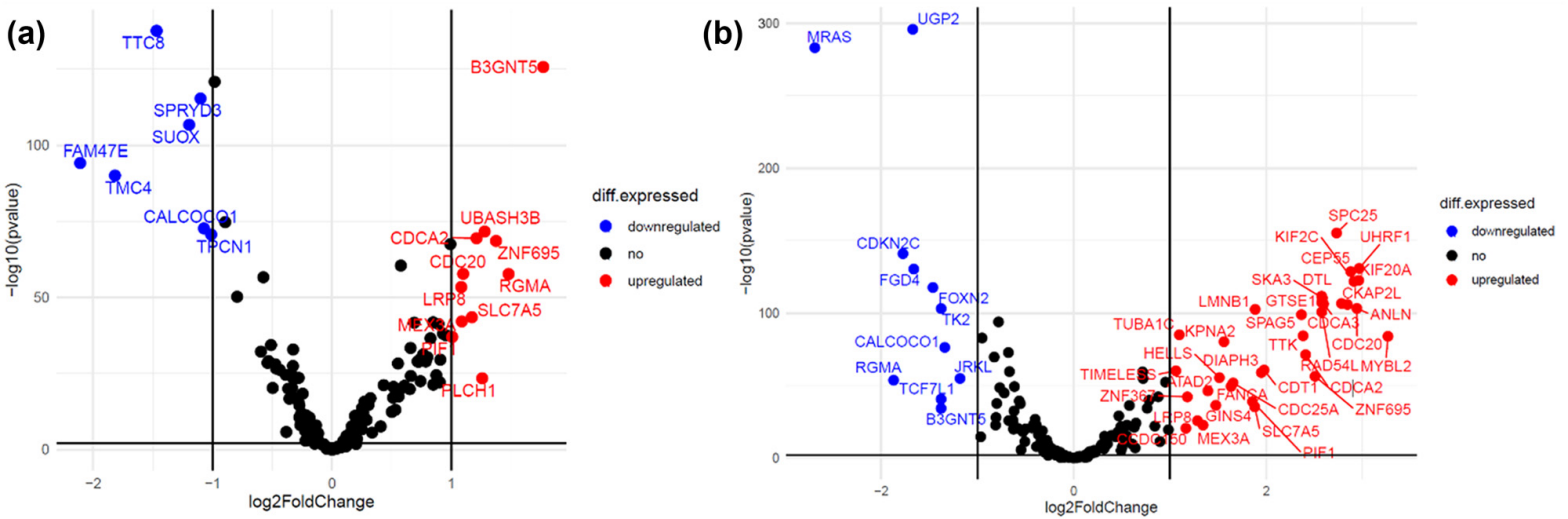
DEA comparison between ER-positive and ER-negative BC patients (a), as well as tumor and normal tissue (b).

DEA was undertaken across two distinct comparative frameworks. First, a comparison between patients with positive and negative ER status was executed, using the latter group as the control cohort. Additionally, a comparison contrasting normal tissue samples against tumor tissue samples was conducted, with the normal samples serving as the reference group. In the initial comparison, *TTC8, SPRYD3, SUOX, FAM47E, TMC4, CALCOCO1,* and *TPCN1* genes were found to be downregulated; whereas *B3GNT5, UBASH3B, CDCA2, CDC20, ZNF695, RGMA, LRP8, SLC7A5, MEX3A, PIF1,* and *PLCH1* displayed a significant upregulation (Figure 4a). As for the normal versus tumor comparison, a collection of genes including *MRAS, UGP2, CDKN2C, FGD4, FOXN2, TK2, CALCOCO1, JRKL, RGMA, TCF7L1*, and *B3GNT5* exhibited downregulation, while a pattern of upregulation was observed for the following genes: *SPC25, KIF2C, UHRF1, CEP55, KIF20A, DTL, SKA3, CKAP2L, ANLN, CDCA3, SPAG5, LMNB1, TTK, RAD54L, MYBL2, CDCA2, KPNA2, TUBA1C, DIAPH3, CDT1, ZNF695, HELLS, TIMELESS, ATAD2, FANCA, GINS4, SLC7A5, PIF1, ZNF367, LRP8,* and *CCDC150* (Figure 4b).

### 3.4 Genes statistically significant for patients’ survival were selected using Kaplan–Meier curves

**Figure 5:**
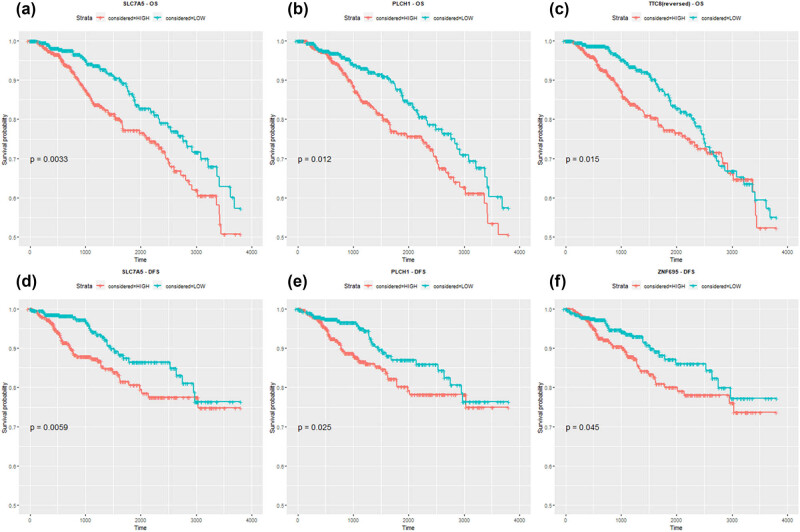
Kaplan–Meier curves for DEGs extracted from ER status comparison ((a) *SLC7A5* [OS], (b) *PLCH1* [OS], (c) *TTC8* [OS], (d) *SLC7A5* [DFS], (e) *PLCH1* [DFS], and (f) *ZNF695* [DFS]) that were found statistically significant.

**Figure 6:**
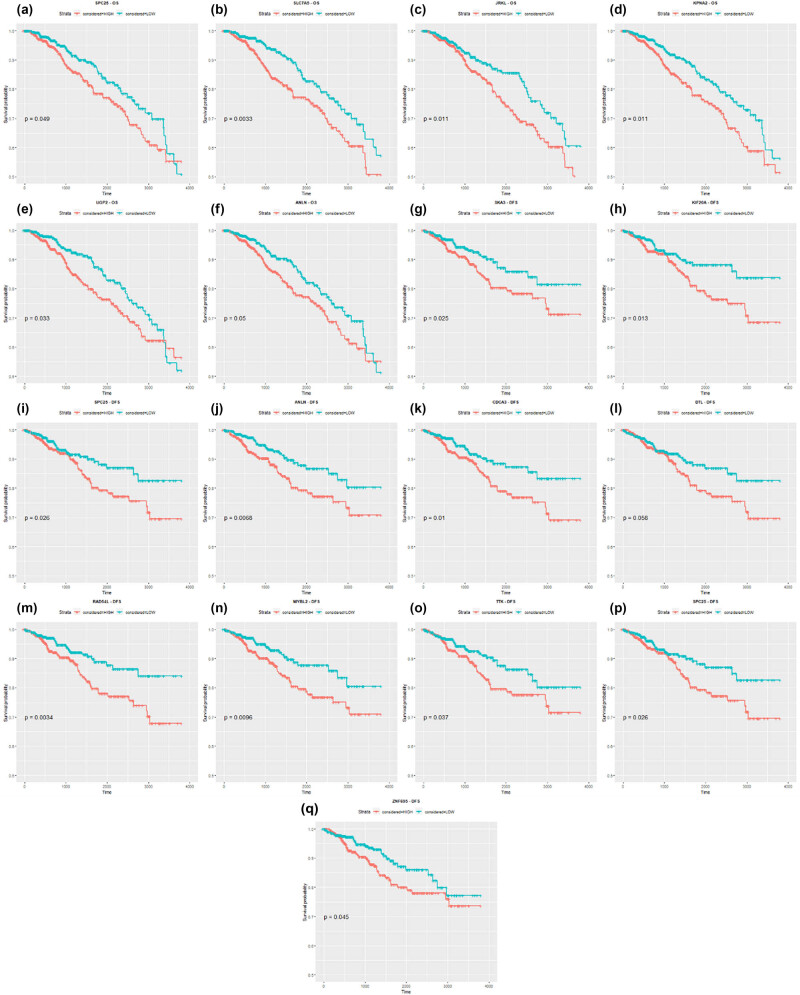
Kaplan–Meier curves for DEGs extracted from tumor and normal tissue comparison in the context of OS (a)–(f) and DFS (g)–(q) ((a) *SPC25*, (b) *SLC7A5*, (c) *JRKL*, (d) *KPNA2*, (e) *UGP2*, (f) *ANLN*, (g) *SKA3*, (h) *KIF20A*, (i) *SPC25*, (j) *ANLN*, (k) *CDCA3*, (l) *DTL*, (m) *RAD54L*, (n) *MYBL2*, (o) *TTK*, (p) *SLC7A5*, and (q) *ZNF695*) that were found statistically significant.

Genes that displayed substantial and statistically significant impact on the survival of these patients were identified and selected for further signature construction. Particularly prominent among these genes in ER+ vs ER− comparison were *TTC8, SLC7A5, PLCH1* (OS), and *ZNF695, SLC7A5, PLCH1* (DFS). For normal vs tumor comparison, the most significant genes included *UGP2, JRKL, SPC25, ANLN, KPNA2, SLC7A5* (OS), as well as *SPC25, KIF20A, SKA3, DTL, CDCA3, ANLN, TTK, RAD54L, MYBL2, ZNF695, SLC7A5* (DFS) (Figures 5 and 6). The *DTL* gene was also included in the list for further evaluation of the gene’s potential impact, as it closely approached statistical significance (*p* = 0.058).

### 3.5 Multi-gene signature construction assessed the cumulative impact on BC patients’ survival

**Figure 7:**
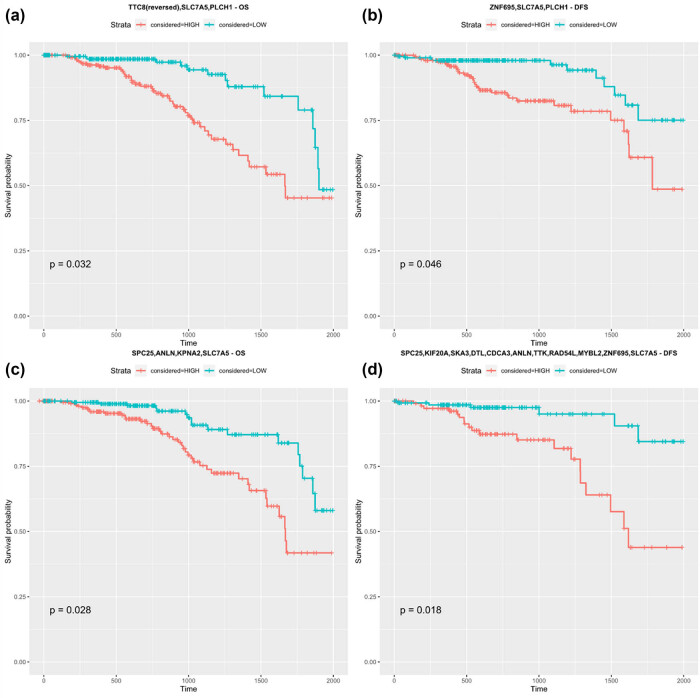
Survival analysis of multi-gene signatures from ER comparisons ((a) OS – *TTC8, SLC5A7, PLCH1* and (b) disease-free survival – *ZNF695, SLC7A5, PLCH1*), and tumor versus normal tissue comparisons ((c) OS – *SPC25, ANLN, KPNA2, SLC7A5* and (d) disease-free survival – *SPC25, KIF20A, SKA3, DTL, CDCA3, ANLN, TTK, RAD54L, MYBL2, ZNF695, SLC7A5*). *TTC8* expression was reversed, as its higher expression was found to be favorable in contrast to other genes.

Gene signatures were constructed on the basis of the prognostic significance of each gene separately. Four novel signatures were analyzed, which included: *TTC8, SLC7A5, PLCH1* (OS, Figure 7a), and *ZNF695, SLC7A5, PLCH*1 (DFS, Figure 7b) from the ER+ vs ER- comparison, as well as *UGP2, JRKL, SPC25, ANLN, KPNA2, SLC7A5* (OS), alongside *SPC25, KIF20A, SKA3, DTL, CDCA3, ANLN, TTK, RAD54L, MYBL2, ZNF695,* and *SLC7A5* (DFS, Figure 7d) from the comparison of tumor vs normal samples. Since the *UGP2, JRKL, SPC25, ANLN, KPNA2, and SLC7A5* signatures with *p* = 0.18 were not statistically significant for the patients’ OS, the genes were rearranged into the most efficient pattern, resulting in the *SPC25, ANLN, KPNA2,* and *SLC7A5* signatures with *p* = 0.028 (Figure 7c). Four sets of genes were further taken into account for binary regression evaluation of the results with ROC curves. The contents of each gene’s signature are summarized in Table S4.

### 3.6 Selected signatures demonstrated predictive capabilities as indicated by ROC curves

**Figure 8:**
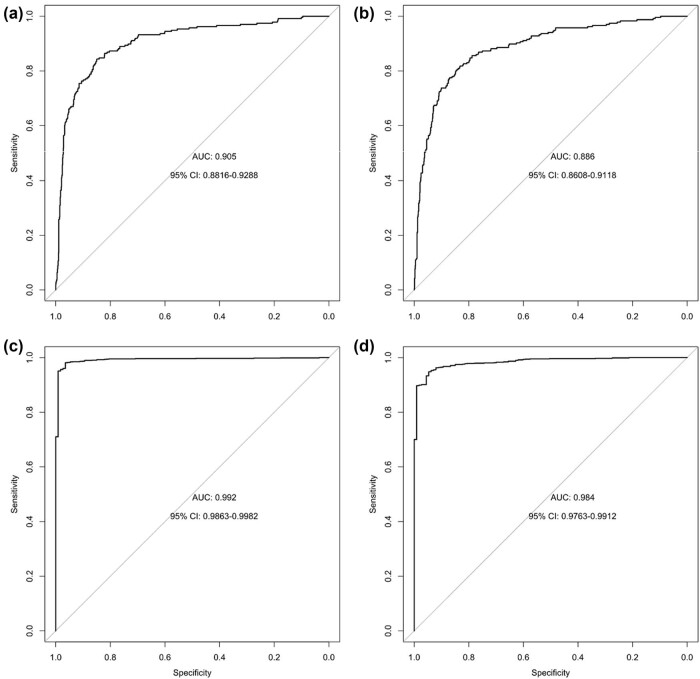
ROC curves for each signature-based binary regression model for ER status comparisons ((a) OS – *TTC8, SLC5A7, PLCH1* and (b) DFS – *ZNF695, SLC7A5, PLCH1*), and tumor versus normal tissue comparisons ((c) OS – *SPC25, ANLN, KPNA2, SLC7A5* and (d) DFS – *SPC25, KIF20A, SKA3, DTL, CDCA3, ANLN, TTK, RAD54L, MYBL2, ZNF695, SLC7A5*).

Predictive properties of multi-gene signatures were evaluated using the binomial logistic regression model, utilizing the collective expression of the chosen sets of genes in conjunction with the ER status (comparison of ER+ and ER− specimens) and the incidence of tumors (comparison between tumor and normal specimens). The resulting AUC values were as follows: 0.905 for OS (Figure 8a) and 0.886 for DFS (Figure 8b), within the ER+ vs ER- signatures (*TTC8*, *SLC7A5*, *PLCH1* and *ZNF695*, *SLC7A5*, *PLCH1*). Similarly, for the normal vs tumor signatures, the corresponding AUC values were 0.992 for OS (Figure 8c) and 0.984 for DFS (Figure 8d) (*SPC25*, *ANLN*, *KPNA2*, *SLC7A5* and *SPC25*, *KIF20A*, *SKA3*, *DTL*, *CDCA3*, *ANLN*, *TTK*, *RAD54L*, *MYBL2*, *ZNF695*, *SLC7A5*). Moreover, the predictive accuracy of the ER-associated signatures reached 88.241% for the OS-based signature and 85.519% for the DFS-based signature. In the case of ‘tumor vs normal’ signatures, the accuracy of prediction achieved notable levels: 97.906% for the OS-based signature and 96.147% for the DFS-based signature.

## 4 Discussion

BC, one of the most common cancers among women, has an annual global incidence of 2.3 million cases. Molecular subtypes, such as luminal A, luminal B, HER2/neu, and triple-negative, guide treatment strategies, with 70% of the cases being hormone-driven, primarily involving ER-expressing luminal subtypes. Estrogens are key drivers in these tumors [2,4,16]. The Wnt signaling pathway, crucial in cell cycle regulation and stem cell renewal, plays a significant role in carcinogenesis and hormone regulation [17,18]. Its dysregulation is linked to developmental defects and cancers. This study explores the Wnt pathway’s regulatory mechanisms to identify potential diagnostic and therapeutic targets for BC [19,20].

The study revealed Wnt gene-regulated processes associated with the ER status in BC patients, primarily involving the cell cycle, DNA replication, and chromatin remodeling, which are also linked with β-catenin/TCF-driven cell cycle activation and estrogen-mediated proliferation [21,22,23]. A relation to the retinoblastoma pathway highlighted *RB1*’s role in tumor suppression, including BC [24]. Additionally, several groups of genes were organized into clusters based on the identified protein–protein interactions. The largest interaction network was identified for proteins encoded by genes such as *AEBP2, BARD1, CDK2, CDC25A, CORO1C, EMG1, EXOSC2, GINS4, KPNA2, LBR, LSM2, MPHOSPH10, PHC1, PHF19, POP1, PSMD12, RCC2, SSRP1, TIMELESS, USP39*, and *ZC3H18*. These proteins are associated with Wnt signaling, which predominantly influences DNA replication, cell cycle progression, and RNA metabolism, as indicated in red in Figure 3. For example, *CDC25A* is a direct target of β-catenin/TCF, as it activates the cell-cycle phosphatase *CDK2*, thus promoting G1/S transition and facilitating β‑catenin transactivation via *PKM2,* which leads to enhanced proliferation [25,26]. Additionally, in BC, *CDK2* and related cyclin–CDK complexes are essential for estrogen-driven cell division, underscoring ER-mediated proliferation [27]. A second network, depicted in blue, is associated with protein localization to the nucleus, apoptosis, and cell cycle, especially its M phase. Comprising genes include *CCT3, KIF20A, LMNB1, LMNB2, NOP2, PHB2, RANBP1, TUBA1C*, and *XPO1*. This network comprises genes that facilitate processes frequently orchestrated also by Wnt signaling and ER activity in many tumors [28,29,30]. Further significant associations were observed for the genes *ANLN, BRIX1, CAD, DDX47, IARS1, ILF2, TUBB*, and *UGP2*, which are highlighted in green. These genes are implicated in the assembly of non-membrane-bound organelles. Among these genes, *ANLN* overexpression was previously found to be correlated with poor survival of BC patients by enhancing cell proliferation and migration, at the same time interacting with Wnt/β‑catenin signaling [31,32]. Finally, the smallest purple network comprises three nodes: *COMMD2, DTL,* and *NAE1*. These genes encode proteins involved in the neddylation process, a type of post-translational modification that adds the ubiquitin-like protein NEDD8 to substrate proteins. Neddylation can influence various biological processes, including carcinogenesis, as it has been found to be upregulated in numerous human cancers [33]. Neddylation inhibitors represent a promising direction for cancer therapy, as they may also promote cancer-related immunosuppression [34]. Notably, in BC, the inhibition of neddylation has been shown to significantly suppress the growth of HER2-positive tumors when combined with trastuzumab [34]. Moreover, the neddylation modification pathway has been activated in breast carcinoma and is associated with ER-α expression [34,35].

In addition, we have successfully extracted a range of gene signatures linked to the Wnt pathway that hold potential as prognostic indicators for individuals afflicted with BC. Existing Wnt-associated biomarkers can be broadly classified based on their function within the pathway, including regulators (e.g., *AXIN2, SFRP1*) [14,36], receptors (e.g., *FZD, LRP* receptor families) [37,38], transcription factors (e.g., *LEF1, TCF4*) [39], or the central mediator of canonical Wnt signaling β-catenin (*CTNNB1*) [40]. Although such classical Wnt targets are already proposed, their expression alone often lacks sufficient specificity or prognostic utility due to the pathway’s various crosstalks and context-dependent activation. In addition, both the Wnt pathway and ER signaling are important drivers of hormone-dependent tumor growth and progression [41,42].

Thus, instead of focusing on single-gene expression patterns among BC patients, multi-gene signatures were developed in this study. Among these, two signatures are intricately tied to the ER status (*TTC8, SLC7A5, PLCH1* – OS; *ZNF695, SLC7A5, PLCH1* – DFS), while another pair is closely associated with the tumor tissue itself (*SPC25, ANLN, KPNA2, SLC7A5* – OS; *SPC25, KIF20A, SKA3, DTL, CDCA3, ANLN, TTK, RAD54L, MYBL2, ZNF695, SLC7A5* – DFS). Among others, is the ER-associated gene *TTC8*, known to play a role in cilia formation [43]. This gene’s capacity to distinguish between luminal (ER+) and non-luminal (ER−) BC cases has been validated by another study conducted by Habashy and colleagues [44]. Moreover, research conducted by Menzl et al. has identified *TTC8* as one of the genes that are commonly downregulated in BC cases, which is in line with our study, as its higher expression was associated with a better prognosis [45]. In our study, however, we have linked the *TTC8* gene with *SLC5A7* (*LAT1*) and *PLCH1*, standing as one of the potential signatures. It has been noted that the *SLC7A5* gene is related to a variety of tumors [46–49]. Regarding BC, *SLC7A5* seems to be especially linked with the aggressive and highly proliferative ER+ subtype, and is also associated with the *MYC* driver gene [46]. The main function of *SLC7A5* involves the import of crucial amino acids into cancer cells, thus making it a viable therapeutic target for cancer management [46,50]. The last gene from this particular signature associated with both ER status and OS of BC patients is *PLCH1*, an enzyme linked with the breakdown of phosphatidylinositol 4,5-bisphosphate [51]. Although recognized as a potentially significant therapeutic target for BC by other studies employing multi-omics data, this gene remains insufficiently studied [52].

In summary, the ER signature, intricately associated with the survival outcomes of BC patients, encompasses *TTC8*, *SLC7A5*, and *PLCH1* genes. This collective set exhibits influence on the OS of BC patients, potentially serving as a combined biomarker panel.

Concerning the ER+ signature linked to DFS, consistency is observed with two of the three notable genes that are also present in the context of OS. Specifically, *SLC7A5* and *PLCH1* maintain their significance, and they are accompanied by the inclusion of *ZNF695*. In a study evaluating KRAB-ZNF factors, *ZNF695* has been found to positively correlate with advanced tumor stage in BC tissues [53]. Another research found that *ZNF695* is associated with the upregulation of several cell cycle genes, mostly in basal-like BC tumors [54]. In addition to ER-associated signatures, analysis of comparisons in normal and tumor samples allowed us to select several genes influencing either OS or DFS. The signature associated with OS that was found significant as a collective set of genes included S*PC25*, *ANLN*, *KPNA2*, and *SLC7A5*. The *SPC25* gene encodes a protein that participates in interactions of the kinetochore microtubule, as well as the activity of the spindle checkpoint. Recently, it has been found to positively correlate with poorer prognosis and survival of BC patients [55,56]. *ANLN*, crucial in cytokinesis and myosin contraction, contributes to immune evasion by cancer cells. Elevated *ANLN* expression is linked to poor cervical cancer survival and serves as a negative prognostic factor. In BC, in silico analysis suggests *ANLN* impacts Th1/Th2 balance in basal and luminal-B subtypes, correlating with poor prognosis [57–59].

Furthermore, another essential gene of this module, *KPNA2*, is found to be an oncogenic factor, being also implicated in nuclear transport [60]. Several studies have identified this gene as a poor prognosis factor for breast and ovarian cancer patients [60–62]. In addition, its expression has also been linked with lower concentrations of DNA repair proteins in the cell nuclei [63]. Taken together, combined *SPC25*, *ANLN*, *KPNA2*, and *SLC7A5* expression seems to be crucial for the OS of individuals with BC. At the same time, each gene of the signature is notably amplified in these patients, in comparison to normal tissue.

The very last signature of the tumor occurrence analysis includes genes such as *SPC25, KIF20A, SKA3, DTL, CDCA3, ANLN, TTK, RAD54L, MYBL2, ZNF695,* and *SLC7A5*, which collectively influence the DFS of BC patients. Several genes have been previously mentioned in connection with other signatures; however, *KIF20A*, *SKA3*, *DTL*, *CDCA3*, *TTK*, *RAD54L*, and *MYBL2* are found to be specifically valuable for DFS, at the same time being significantly overexpressed in cancer tissue. *KIF20A* is a gene encoding a cytokinesis-related protein [64]. The current literature confirms that in ER+ cases of BC, the gene has been found to serve as an independent prognostic factor [65,66]. Its expression has also been linked with poorer prognosis of such patients, indicating a potential to be a therapeutic target as well [65]. Next, *SKA3* encodes a protein that is a part of a larger complex, functioning during mitosis via microtubule attachment to the kinetochores [67]. The gene’s overexpression has been linked with promoting the growth of BC, and thus, is associated with a worse survival ratio in this group of patients. This is due to the regulation of *PLK-1*, which is also involved in eukaryotic cell division, as its expression is frequently elevated in certain tumors [68–71]. Another component of the signature, *DTL*, is also associated with cells [72]. It has a vital role in cancer progression by taking part in the PDCD4 protein degradation, which is known to be a tumor suppressor influencing programmed cell death [73]. *DTL*’s elevated expression is often associated with lower survival rates of various cancer patients, thus proving the gene’s clinical value [74–77]. *CDCA3* is another gene of the signature associated with cell multiplication, being one of the pivotal regulators of cell mitosis [78]. The current literature reports that inhibiting *CDCA3* expression might be of great importance in neoplastic proliferation suppression [79,80]. *TTK* encodes a kinase involved in mitosis and cell proliferation, playing a role in neoplastic development. Its inhibition enhances radiosensitivity in basal-like BC. Although often elevated in TNBC, *TTK* was identified as a positive prognostic biomarker for this type of cancer [81–83].

Next, *RAD54L* is a member of the DEAD-like helicase superfamily and is implicated in homologous recombination and repair of DNA [84]. The current literature confirms its association with carcinogenesis, e.g., by promoting progression or influencing repair mechanisms in various cancers [85,86]. Also, *RAD45L* expression is reported to be upregulated in BC and other neoplasms [87]. *MYBL2*, a proto-oncogene involved in *de novo* purine synthesis, regulates the cell cycle and is linked to poor prognosis in tumors. It promotes neoplastic proliferation and metastasis via *CDCA3* activation. Frequently overexpressed in aggressive BCs like TNBC, *MYBL2* is a potential therapeutic target [88–90].

Multiple genes included in our signatures have been previously reported as potential biomarkers in various cancers. However, the specific roles of several signature genes in BC remain poorly understood. Thus, the constructed signatures do not replace classical markers but rather expand the landscape of Wnt and ER-related prognostic tools in BC by incorporating less characterized effectors. In addition, such panels may better capture the molecular heterogeneity of BC [91–93].

Table 1 describes the characteristics of each identified gene to summarize the review of signature-based genes.

**Table 1:** Summary of genes included in at least one signature for BC patients provided with favorable expression status, short functional annotations, and experimental validation summary

Gene symbol	Favorable expression status	Name and summarized function of the protein [94]	Experimental validation in the literature
*ANLN*	Low	Actin-binding protein that participates in cell growth, migration, and cytokinesis	Often overexpressed in tumors; strongly correlated with poor prognosis [95–97]
*CDCA3*	Low	F-box-like protein essential for mitosis initiation	Found overexpressed in tumors and cell lines; linked with poor prognosis [80,98]
*DTL*	Low	Denticleless E3 ubiquitin protein ligase homolog associated with cell cycle regulation, responds to DNA damage, and translesion DNA synthesis	Found overexpressed in tumors, while high expression predicts worse survival ratio [99,100]
*KIF20A*	Low	Kinesin family member 20A is engaged in the formation of microtubule bundles, midbody abscission, and the regulation of cytokinesis	Found overexpressed in tumors, knockdown reduces cell proliferation, and is linked with poor prognosis [65,101]
*MYBL2*	Low	Myb-related protein B is a transcription factor involved in cell cycle progression	Overexpressed in tumors and cell lines, and high expression linked with poor prognosis; in BC cell models, inhibition of *MYBL2* was found to reduce proliferation [102–104]
*PLCH1*	Low	Phospholipase C Eta 1 is an intracellular enzyme associated with lipid metabolism, particularly lipid degradation	Overexpressed in tumors; knockdown in cell lines inhibits cell proliferation [105]
*RAD54L*	Low	RAD54-like protein plays an essential role in the homologous recombination of the DNA double-strand breaks pathway	High expression predicts poor prognosis; cell line studies show regulation of DNA repair dynamics; mostly supported only by expression and pan-cancer data [106,107]
*SKA3*	Low	Spindle and kinetochore-associated protein 3; component of the outer kinetochore complex responsible for proper chromosome segregation	Found overexpressed in tumors; high expression predicts poor prognosis and is associated with ER/PR status [68,108]
*SLC7A5*	Low	Solute carrier family 7 member 5, taking part in amino-acid transport; possibly mediates the transport of thyroid hormones	Overexpressed in BC tissues; expression is the highest in more aggressive BRCA subtypes; supports growth and survival of rapidly dividing cancer cells [46,109]
*SPC25*	Low	SPC25 component of the NDC80 kinetochore complex is essential for chromosome segregation and spindle checkpoint activity	Found overexpressed in tumors; high expression linked to advanced stage, higher grade, and poor prognosis [55,110]
*TTC8*	High	Tetratricopeptide repeat domain 8 is associated with cilium biogenesis and degradation, with a function related to protein transportation	Potential mechanistic links to Wnt signaling or cancer progression remain speculative and mostly characterized by bioinformatic and expression data [45]
*TTK*	Low	Monopolar spindle 1 (Mps1) kinase phosphorylates proteins on serine, threonine, and tyrosine, and is possibly related to cell proliferation	Found overexpressed in breast tumors, high expression correlates with poor prognosis and enhanced tumor aggressiveness; inhibition in cell lines suppresses neoplastic growth; a promising candidate for radiosensitizing strategies for patients with basal-like BC [111,112]
*ZNF695*	Low	Zinc finger protein 695 is a transcription factor that potentially facilitates the activity of DNA-binding transcription factors	Found overexpressed in breast tumors and cell lines; associated with BRCA molecular subtypes; the highest expression found in aggressive subtypes; multiple alternatively spliced variants detected in BC cells; no direct functional studies reported [53,54,113]

## 5 Conclusions

The Wnt pathway plays a crucial role in BC progression, firmly linked to the ER status. Ontological analysis of its components revealed their involvement in cell cycle regulation, RNA metabolism, membraneless organelle assembly, and neddylation. The study identified four novel prognostic signatures influencing BC patients’ survival: two differentiate ER-positive from ER-negative cases, and two distinguish tumor from normal tissues with high accuracy. Studies have supported the relevance of some genes in these signatures, such as *ANLN, CDCA3, KIF20A, SKA3, SLC7A5,* and *TTK.* However, multiple genes included in our signatures, such as *DTL, MYBL2*, *PLCH1, RAD54L, SPC25, TTC8,* and *ZNF695*, are either poorly characterized or have not been previously described in the context of BC, making them promising candidates for further investigation. Overall, genes included in the signatures have been proven to have clinical value and could contribute to improved diagnosis and prognosis of BC patients. In the future, such panels may also serve as novel sets of therapeutic targets for further management of malignancies, possibly being more effective in particular treatments than a single factor.

## Supplementary Material

Supplementary Table

## References

[1] Lei S, Zheng R, Zhang S, Wang S, Chen R, Sun K, et al. Global patterns of breast cancer incidence and mortality: A population-based cancer registry data analysis from 2000 to 2020. Cancer Commun. 2021;41(11):1183. 10.1002/cac2.12207 PMC8626596 34399040

[2] Łukasiewicz S, Czeczelewski M, Forma A, Baj J, Sitarz R, Stanisławek A. Breast cancer – epidemiology, risk factors, classification, prognostic markers, and current treatment strategies – an updated review. Cancers (Basel). 2021;13(17). 10.3390/cancers13174287 PMC8428369 34503097

[3] Sung H, Ferlay J, Siegel RL, Laversanne M, Soerjomataram I, Jemal A, et al. Global cancer statistics 2020: GLOBOCAN estimates of incidence and mortality worldwide for 36 cancers in 185 countries. CA Cancer J Clin. 2021;71(3):209–49. 10.3322/caac.21660 33538338

[4] Al-thoubaity FK. Molecular classification of breast cancer: A retrospective cohort study. Ann Med Surg. 2020;49:44. 10.1016/j.amsu.2019.11.021 PMC6926136 31890196

[5] Fragomeni SM, Sciallis A, Jeruss JS. Molecular subtypes and local-regional control of breast cancer. Surg Oncol Clin N Am. 2018;27(1):95. 10.1016/j.soc.2017.08.005 PMC5715810 29132568

[6] Nounou MI, Elamrawy F, Ahmed N, Abdelraouf K, Goda S, Syed-Sha-Qhattal H. Breast cancer: conventional diagnosis and treatment modalities and recent patents and technologies. Breast Cancer (Auckl). 2015;9(Suppl 2):17. 10.4137/BCBCR.S29420 PMC4589089 26462242

[7] Cheung KL. Treatment strategies and survival outcomes in breast cancer. Cancers (Basel). 2020;12(3):735. 10.3390/cancers12030735 PMC7140050 32244985

[8] Wang J, Wu SG. Breast cancer: an overview of current therapeutic strategies, challenge, and perspectives. Breast Cancer: Targets Ther. 2023;15:721. 10.2147/BCTT.S432526 PMC10596062 37881514

[9] Ginsburg O, Yip CH, Brooks A, Cabanes A, Caleffi M, Yataco JAD, et al. Breast cancer early detection: a phased approach to implementation. Cancer. 2020;126(Suppl 10):2379. 10.1002/cncr.32887 PMC7237065 32348566

[10] DeLouize AM, Eick G, Karam SD, Snodgrass JJ. Current and future applications of biomarkers in samples collected through minimally invasive methods for cancer medicine and population-based research. Am J Hum Biol. 2022;34(11):e23665. 10.1002/ajhb.23665 PMC9894104 34374148

[11] Yin P, Wang W, Zhang Z, Bai Y, Gao J, Zhao C. Wnt signaling in human and mouse breast cancer: Focusing on Wnt ligands, receptors and antagonists. Cancer Sci. 2018;109(11):3368. 10.1111/cas.13771 PMC6215866 30137666

[12] Komiya Y, Habas R. Wnt signal transduction pathways. Organogenesis. 2008;4(2):68. 10.4161/org.4.2.5851 PMC2634250 19279717

[13] Turashvili G, Bouchal J, Burkadze G, Kolar Z. Wnt signaling pathway in mammary gland development and carcinogenesis. Pathobiology. 2007;73(5):213–23. 10.1159/000098207 17314492

[14] Xu X, Zhang M, Xu F, Jiang S. Wnt signaling in breast cancer: biological mechanisms, challenges and opportunities. Mol Cancer. 2020;19(1):165. 10.1186/s12943-020-01276-5 PMC7686704 33234169

[15] Zhang B, Horvath S. A general framework for weighted gene co-expression network analysis. Stat Appl Genet Mol Biol. 2005;4:17. 10.2202/1544-6115.1128 16646834

[16] Clusan L, Ferrière F, Flouriot G, Pakdel F. A basic review on estrogen receptor signaling pathways in breast cancer. Int J Mol Sci. 2023;24(7):6834. 10.3390/ijms24076834 PMC10095386 37047814

[17] Abreu de Oliveira WA, El Laithy Y, Bruna A, Annibali D, Lluis F. Wnt signaling in the breast: from development to disease. Front Cell Dev Biol. 2022;10:884467. 10.3389/fcell.2022.884467 PMC9157790 35663403

[18] Chen Y, Chen Z, Tang Y, Xiao Q. The involvement of noncanonical Wnt signaling in cancers. Biomed Pharmacother. 2021;133:110946. 10.1016/j.biopha.2020.110946 33212376

[19] Zhan T, Rindtorff N, Boutros M. Wnt signaling in cancer. Oncogene. 2017;36(11):1461–73. 10.1038/onc.2016.304 PMC5357762 27617575

[20] Duchartre Y, Kim YM, Kahn M. The Wnt signaling pathway in cancer. Crit Rev Oncol Hematol. 2016;99:141. 10.1016/j.critrevonc.2015.12.005 PMC5853106 26775730

[21] Zhang X, Yu X. Crosstalk between Wnt/β-catenin signaling pathway and DNA damage response in cancer: a new direction for overcoming therapy resistance. Front Pharmacol. 2023;14:1230822. 10.3389/fphar.2023.1230822 PMC10433774 37601042

[22] Mora-Blanco EL, Mishina Y, Tillman EJ, Cho YJ, Thom CS, Pomeroy SL, et al. Activation of β-catenin/TCF targets following loss of the tumor suppressor SNF5. Oncogene. 2013;33(7):933. 10.1038/onc.2013.37 PMC4893026 23435428

[23] Hewitt SC, Li Y, Li L, Korach KS. Estrogen-mediated regulation of Igf1 transcription and uterine growth involves direct binding of estrogen receptor α to estrogen-responsive elements. J Biol Chem. 2009;285(4):2676. 10.1074/jbc.M109.043471 PMC2807324 19920132

[24] Yun J, Li Y, Xu CT, Pan BR. Epidemiology and Rb1 gene of retinoblastoma. Int J Ophthalmol. 2011;4(1):103. 10.3980/j.issn.2222-3959.2011.01.24 PMC3340672 22553621

[25] Vijayakumar S, Liu G, Rus IA, Yao S, Chen Y, Akiri G, et al. Wnt signaling is activated at high frequency and drives proliferation of multiple human sarcoma subtypes through a TCF/β-catenin target gene, CDC25A. Cancer Cell. 2011;19(5):601. 10.1016/j.ccr.2011.03.010 PMC3116447 21575861

[26] Mukherjee J, Ohba S, See WL, Phillips JJ, Molinaro AM, Pieper RO. PKM2 uses control of HuR localization to regulate p27 and cell cycle progression in human glioblastoma cells. Int J Cancer. 2016;139(1):99. 10.1002/ijc.30041 PMC6615049 26874904

[27] Ding L, Cao J, Lin W, Chen H, Xiong X, Ao H, et al. The roles of cyclin-dependent kinases in cell-cycle progression and therapeutic strategies in human breast cancer. Int J Mol Sci. 2020;21(6):1960. 10.3390/ijms21061960 PMC7139603 32183020

[28] Liu W, Lu Y, Yan X, Lu Q, Sun Y, Wan X, et al. Current understanding on the role of CCT3 in cancer research. Front Oncol. 2022;12:961733. 10.3389/fonc.2022.961733 PMC9520704 36185198

[29] Boonekamp KE, Heo I, Artegiani B, Asra P, van Son G, de Ligt J, et al. Identification of novel human Wnt target genes using adult endodermal tissue-derived organoids. Dev Biol. 2021;474:37–47. 10.1016/j.ydbio.2021.01.009 33571486

[30] Bell ES, Shah P, Zuela-Sopilniak N, Kim D, Varlet AA, Morival JLP, et al. Low lamin A levels enhance confined cell migration and metastatic capacity in breast cancer. Oncogene. 2022;41(36):4211–30. 10.1038/s41388-022-02420-9 PMC9925375 35896617

[31] Pandi NS, Manimuthu M, Harunipriya P, Murugesan M, Asha GV, Rajendran S. In silico analysis of expression pattern of a Wnt/β-catenin responsive gene ANLN in gastric cancer. Gene. 2014;545(1):23–9. 10.1016/j.gene.2014.05.013 24809965

[32] Zhu X, Zhang Y, Bian R, Zhu J, Shi W, Ye Y. ANLN promotes the proliferation and migration of gallbladder cancer cells via STRA6-mediated activation of PI3K/AKT signaling. Cancers (Basel). 2024;16(4):752. 10.3390/cancers16040752 PMC10887181 38398143

[33] Zhou L, Jiang Y, Luo Q, Li L, Jia L. Neddylation: A novel modulator of the tumor microenvironment. Mol Cancer. 2019;18(1):1–11. 10.1186/s12943-019-0979-1 PMC6446326 30943988

[34] Xia X, Hu T, He X, Liu Y, Yu C, Kong W, et al. Neddylation of HER2 inhibits its protein degradation and promotes breast cancer progression. Int J Biol Sci. 2023;19(2):377–92. 10.7150/ijbs.75852 PMC9830515 36632463

[35] Jia X, Li C, Li L, Liu X, Zhou L, Zhang W, et al. Neddylation inactivation facilitates FOXO3a nuclear export to suppress estrogen receptor transcription and improve fulvestrant sensitivity. Clin Cancer Res. 2019;25(12):3658–72. 10.1158/1078-0432.CCR-18-2434 30833270

[36] Tufail M, Jiang CH, Li N. Wnt signaling in cancer: from biomarkers to targeted therapies and clinical translation. Mol Cancer. 2025;24(1):107. 10.1186/s12943-025-02306-w PMC11963613 40170063

[37] Martinez-Marin D, Stroman GC, Fulton CJ, Pruitt K. Frizzled receptors: gatekeepers of Wnt signaling in development and disease. Front Cell Dev Biol. 2025;13:1599355. 10.3389/fcell.2025.1599355 PMC12078226 40376615

[38] Liu CC, Prior J, Piwnica-Worms D, Bu G. LRP6 overexpression defines a class of breast cancer subtype and is a target for therapy. Proc Natl Acad Sci U S A. 2010;107(11):5136. 10.1073/pnas.0911220107 PMC2841938 20194742

[39] Lima BM, Azevedo ALKde, Giner IS, Gomig THB, Ribeiro EMdeSF, Cavalli IJ. Biomarker potential of the LEF1/TCF family members in breast cancer: Bioinformatic investigation on expression and clinical significance. Genet Mol Biol. 2023;46(4):e20220346. 10.1590/1678-4685-GMB-2022-0346 PMC10723634 38100720

[40] van Schie EH, van Amerongen R. Aberrant WNT/CTNNB1 signaling as a therapeutic target in human breast cancer: weighing the evidence. Front Cell Dev Biol. 2020;8:25. 10.3389/fcell.2020.00025 PMC7005411 32083079

[41] Mastroianni M, Kim S, Kim YC, Esch A, Wagner C, Alexander CM. Wnt signaling can substitute for estrogen to induce division of ERα-positive cells in a mouse mammary tumor model. Cancer Lett. 2009;289(1):23. 10.1016/j.canlet.2009.07.012 PMC2874254 19665836

[42] Parsons MJ, Tammela T, Dow LE. WNT as a driver and dependency in cancer. Cancer Discov. 2021;11(10):2413. 10.1158/2159-8290.CD-21-0190 PMC8487948 34518209

[43] Chen HY, Kelley RA, Li T, Swaroop A. Primary cilia biogenesis and associated retinal ciliopathies. Semin Cell Dev Biol. 2021;110:70. 10.1016/j.semcdb.2020.07.013 PMC7855621 32747192

[44] Habashy HO, Powe DG, Glaab E, Ball G, Spiteri I, Krasnogor N, et al. RERG (Ras-like, oestrogen-regulated, growth-inhibitor) expression in breast cancer: a marker of ER-positive luminal-like subtype. Breast Cancer Res Treat. 2010;128(2):315–26. 10.1007/s10549-010-1073-y 20697807

[45] Menzl I, Lebeau L, Pandey R, Hassounah NB, Li FW, Nagle R, et al. Loss of primary cilia occurs early in breast cancer development. Cilia. 2014;3(1):1–17. 10.1186/2046-2530-3-7 PMC4076761 24987519

[46] El Ansari R, Craze ML, Miligy I, Diez-Rodriguez M, Nolan CC, Ellis IO, et al. The amino acid transporter SLC7A5 confers a poor prognosis in the highly proliferative breast cancer subtypes and is a key therapeutic target in luminal B tumours. Breast Cancer Res. 2018;20(1):1–17. 10.1186/s13058-018-0946-6 PMC5863851 29566741

[47] Kobayashi H, Ishii Y, Takayama T. Expression of L-type amino acid transporter 1 (LAT1) in esophageal carcinoma. J Surg Oncol. 2005;90(4):233–8. 10.1002/jso.20257 15906366

[48] Jung HY, In JK, Kim H, Kim HJ, Moon JJ, Sang GA, et al. Amino acid transport system L is differently expressed in human normal oral keratinocytes and human oral cancer cells. Cancer Lett. 2005;222(2):237–45. 10.1016/j.canlet.2004.09.040 15863273

[49] Nakanishi K, Matsuo H, Kanai Y, Endou H, Hiroi S, Tominaga S, et al. LAT1 expression in normal lung and in atypical adenomatous hyperplasia and adenocarcinoma of the lung. Virchows Arch. 2006;448(2):142–50. 10.1007/s00428-005-0063-7 16175382

[50] Kanai Y. Amino acid transporter LAT1 (SLC7A5) as a molecular target for cancer diagnosis and therapeutics. Pharmacol Ther. 2022;230:107964. 10.1016/j.pharmthera.2021.107964 34390745

[51] Joensuu M, Wallis TP, Saber SH, Meunier FA. Phospholipases in neuronal function: A role in learning and memory? J Neurochem. 2020;153(3):300–33. 10.1111/jnc.14918 31745996

[52] Barupal DK, Gao B, Budczies J, Phinney BS, Perroud B, Denkert C, et al. Prioritization of metabolic genes as novel therapeutic targets in estrogen-receptor negative breast tumors using multi-omics data and text mining. Oncotarget. 2019;10(39):3894. 10.18632/oncotarget.26995 PMC6570467 31231467

[53] Machnik M, Cylwa R, Kiełczewski K, Biecek P, Liloglou T, Mackiewicz A, et al. The expression signature of cancer-associated KRAB-ZNF factors identified in TCGA pan‐cancer transcriptomic data. Mol Oncol. 2019;13(4):701. 10.1002/1878-0261.12407 PMC6442004 30444046

[54] Li R, Campos J, Iida J. A gene regulatory program in human breast cancer. Genetics. 2015;201(4):1341–8. 10.1534/genetics.115.180125 PMC4676531 26510790

[55] Wang Q, Zhu Y, Li Z, Bu Q, Sun T, Wang H, et al. Up-regulation of SPC25 promotes breast cancer. Aging (Albany NY). 2019;11(15):5689. 10.18632/aging.102153 PMC6710047 31400751

[56] Deng N, Chen K, Fan H, Jin F. The synergistic effect of CDKN2B-AS1 and SPC25 on triple-negative breast cancer. Ann Transl Med. 2022;10(14):783. 10.21037/atm-22-2900 PMC9372695 35965791

[57] Liu K, Cui L, Li C, Tang C, Niu Y, Hao J, et al. Pan-cancer analysis of the prognostic and immunological role of ANLN: An onco-immunological biomarker. Front Genet. 2022;13:922472. 10.3389/fgene.2022.922472 PMC9390797 35991576

[58] Xia L, Su X, Shen J, Meng Q, Yan J, Zhang C, et al. ANLN functions as a key candidate gene in cervical cancer as determined by integrated bioinformatic analysis. Cancer Manag Res. 2018;10:663. 10.2147/CMAR.S162813 PMC5896649 29670400

[59] Xiao Y, Deng Z, Li Y, Wei B, Chen X, Zhao Z, et al. ANLN and UBE2T are prognostic biomarkers associated with immune regulation in breast cancer: a bioinformatics analysis. Cancer Cell Int. 2022;22(1):193. 10.1186/s12935-022-02611-0 PMC9109316 35578283

[60] Alshareeda AT, Negm OH, Green AR, Nolan CC, Tighe P, Albarakati N, et al. KPNA2 is a nuclear export protein that contributes to aberrant localisation of key proteins and poor prognosis of breast cancer. Br J Cancer. 2015;112(12):1929. 10.1038/bjc.2015.165 PMC4580386 25989275

[61] Huang L, Wang HY, Li JD, Wang JH, Zhou Y, Luo RZ, et al. KPNA2 promotes cell proliferation and tumorigenicity in epithelial ovarian carcinoma through upregulation of c-Myc and downregulation of FOXO3a. Cell Death Dis. 2013;4(8):e745. 10.1038/cddis.2013.256 PMC3763430 23907459

[62] Cui X, Jing X, Wu X, Xu J, Liu Z, Huo K, et al. Analyses of DNA methylation involved in the activation of nuclear karyopherin alpha 2 leading to identify the progression and prognostic significance across human breast cancer. Cancer Manag Res. 2020;12:6665. 10.2147/CMAR.S261290 PMC7416187 32801900

[63] Alnoumas L, van den Driest L, Apczynski Z, Lannigan A, Johnson CH, Rattray NJW, et al. Evaluation of the role of KPNA2 mutations in breast cancer prognosis using bioinformatics datasets. BMC Cancer. 2022;22(1):1–11. 10.1186/s12885-022-09969-4 PMC9364282 35948941

[64] Jin Z, Peng F, Zhang C, Tao S, Xu D, Zhu Z. Expression, regulating mechanism and therapeutic target of KIF20A in multiple cancer. Heliyon. 2023;9(2):e13195. 10.1016/j.heliyon.2023.e13195 PMC9925975 36798768

[65] Nakamura M, Takano A, Thang PM, Tsevegjav B, Zhu M, Yokose T, et al. Characterization of KIF20A as a prognostic biomarker and therapeutic target for different subtypes of breast cancer. Int J Oncol. 2020;57(1):277–88. 10.3892/ijo.2020.5060 32467984

[66] Huang X, Li S, Gao W, Shi J, Cheng M, Mi Y, et al. KIF20A is a prognostic marker for female patients with estrogen receptor-positive breast cancer and receiving tamoxifen as adjuvant endocrine therapy. Int J Gen Med. 2023;16:3623–35. 10.2147/IJGM.S425918 PMC10455948 37637711

[67] Gaitanos TN, Santamaria A, Jeyaprakash AA, Wang B, Conti E, Nigg EA. Stable kinetochore-microtubule interactions depend on the Ska complex and its new component Ska3/C13Orf3. EMBO J. 2009;28(10):1442–52. 10.1038/emboj.2009.96 PMC2669960 19360002

[68] Zhong Y, Zhuang Z, Mo P, Lin M, Gong J, Huang J, et al. Overexpression of SKA3 correlates with poor prognosis in female early breast cancer. PeerJ. 2021;9:e12506. 10.7717/peerj.12506 PMC8675262 34993016

[69] Ruan LW, Li PP, Jin LP. SKA3 promotes cell growth in breast cancer by inhibiting PLK-1 protein degradation. Technol Cancer Res Treat. 2020;19:1533033820947488. 10.1177/1533033820947488 PMC7436789 32799774

[70] Lee SY, Jang C, Lee KA. Polo-like kinases (Plks), a key regulator of cell cycle and new potential target for cancer therapy. Dev Reprod. 2014;18(1):65. 10.12717/DR.2014.18.1.065 PMC4282265 25949173

[71] Liu Z, Sun Q, Wang X. PLK1, a potential target for cancer therapy. Transl Oncol. 2017;10(1):22. 10.1016/j.tranon.2016.10.003 PMC5124362 27888710

[72] Sansam CL, Shepard JL, Lai K, Ianari A, Danielian PS, Amsterdam A, et al. DTL/CDT2 is essential for both CDT1 regulation and the early G2/M checkpoint. Genes Dev. 2006;20(22):3117. 10.1101/gad.1482106 PMC1635147 17085480

[73] Cui H, Wang Q, Lei Z, Feng M, Zhao Z, Wang Y, et al. DTL promotes cancer progression by PDCD4 ubiquitin-dependent degradation. J Exp Clin Cancer Res. 2019;38(1):1–13. 10.1186/s13046-019-1358-x PMC6693180 31409387

[74] Luo Y, He Z, Liu W, Zhou F, Liu T, Wang G. DTL is a prognostic biomarker and promotes bladder cancer progression through regulating the AKT/mTOR axis. Oxid Med Cell Longev. 2022;2022:3369858. 10.1155/2022/3369858 PMC8799954 35103094

[75] Li Z, Wang R, Qiu C, Cao C, Zhang J, Ge J, et al. Role of DTL in hepatocellular carcinoma and its impact on the tumor microenvironment. Front Immunol. 2022;13:834606. 10.3389/fimmu.2022.834606 PMC8980229 35392073

[76] Liu S, Gu L, Wu N, Song J, Yan J, Yang S, et al. Overexpression of DTL enhances cell motility and promotes tumor metastasis in cervical adenocarcinoma by inducing RAC1-JNK-FOXO1 axis. Cell Death Dis. 2021;12(10):1–9. 10.1038/s41419-021-04179-5 PMC8505428 34635635

[77] Tang Y, Lei Y, Gao P, Jia J, Du H, Wang Q, et al. Pan-cancer analysis and experimental validation of DTL as a potential diagnosis, prognosis and immunotherapy biomarker. BMC Cancer. 2023;23(1):1–20. 10.1186/s12885-023-10755-z PMC10088150 37038185

[78] Zhang W, Lu Y, Li X, Zhang J, Zheng L, Zhang W, et al. CDCA3 promotes cell proliferation by activating the NF-κB/cyclin D1 signaling pathway in colorectal cancer. Biochem Biophys Res Commun. 2018;500:196–203. 10.1016/j.bbrc.2018.04.034 29627567

[79] Yang H, Wei X, Zhang L, Xiang L, Wang P. Pan-cancer analysis identifies CDCA3 as a novel prognostic marker associated with immune infiltration in lung adenocarcinoma through bioinformatics analysis. Transl Cancer Res. 2022;11(8):2902–16. 10.21037/tcr-22-1901 PMC9459646 36093552

[80] Shen D, Fang Y, Zhou F, Deng Z, Qian K, Wang G, et al. The inhibitory effect of silencing CDCA3 on migration and proliferation in bladder urothelial carcinoma. Cancer Cell Int. 2021;21(1):1–14. 10.1186/s12935-021-01969-x PMC8114508 33980246

[81] Dou Z, Ding X, Zereshki A, Zhang Y, Zhang J, Wang F, et al. TTK kinase is essential for the centrosomal localization of TACC2. FEBS Lett. 2004;572(1–3):51–6. 10.1016/j.febslet.2004.06.092 15304323

[82] Huang H, Yang Y, Zhang W, Liu X, Yang G. TTK regulates proliferation and apoptosis of gastric cancer cells through the Akt-mTOR pathway. FEBS Open Bio. 2020;10(8):1542. 10.1002/2211-5463.12909 PMC7396433 32530571

[83] Xu Q, Xu Y, Pan B, Wu L, Ren X, Zhou Y, et al. TTK is a favorable prognostic biomarker for triple-negative breast cancer survival. Oncotarget. 2016;7(49):81815. 10.18632/oncotarget.13245 PMC5348432 27833085

[84] Li D, Liu H, Jiao L, Chang DZ, Beinart G, Wolff RA, et al. Significant impact of homologous recombination DNA repair gene polymorphisms on pancreatic cancer survival. Cancer Res. 2006;66(6):3323. 10.1158/0008-5472.CAN-05-3032 PMC1462866 16540687

[85] Wang Y, Zhou T, Chen H, Wen S, Dao P, Chen M. Rad54L promotes bladder cancer progression by regulating cell cycle and cell senescence. Medical Oncol. 2022;39:1–11. 10.1007/s12032-022-01751-7 36071250

[86] Zheng S, Yao L, Li F, Huang L, Yu Y, Lin Z, et al. Homologous recombination repair rathway and RAD54L in early-stage lung adenocarcinoma. PeerJ. 2021;9:e10680. 10.7717/peerj.10680 PMC7894105 33628633

[87] Bong IPN, Ng CC, Othman N, Esa E. Gene expression profiling and in vitro functional studies reveal RAD54L as a potential therapeutic target in multiple myeloma. Genes Genom. 2022;44(8):957. 10.1007/s13258-022-01272-7 PMC9273556 35689754

[88] Musa J, Aynaud MM, Mirabeau O, Delattre O, Grünewald TGP. MYBL2 (B-Myb): a central regulator of cell proliferation, cell survival and differentiation involved in tumorigenesis. Cell Death Dis. 2017;8(6):e2895. 10.1038/cddis.2017.244 PMC5520903 28640249

[89] Liu W, Shen D, Ju L, Zhang R, Du W, Jin W, et al. MYBL2 promotes proliferation and metastasis of bladder cancer through transactivation of CDCA3. Oncogene. 2022;41(41):4606–17. 10.1038/s41388-022-02456-x 36071275

[90] Bayley R, Ward C, Garcia P. MYBL2 amplification in breast cancer: Molecular mechanisms and therapeutic potential. Biochim Biophys Acta Rev Cancer. 2020;1874(2):188407. 10.1016/j.bbcan.2020.188407 32853735

[91] Licata L, Cosentini D, De Sanctis R, Iorfida M, Caremoli ER, Vingiani A, et al. Multigene signatures for early breast cancer in clinical practice: A report of the Lombardy genomic assays for breast cancer working group. Front Oncol. 2023;13:1081885. 10.3389/fonc.2023.1081885 PMC10025563 36950554

[92] Poudel P, Nyamundanda G, Patil Y, Cheang MCU, Sadanandam A. Heterocellular gene signatures reveal luminal-A breast cancer heterogeneity and differential therapeutic responses. NPJ Breast Cancer. 2019;5(2):21. 10.1038/s41523-019-0116-8 PMC6677833 31396557

[93] Rivenbark AG, O’Connor SM, Coleman WB. Molecular and cellular heterogeneity in breast cancer: Challenges for personalized medicine. Am J Pathol. 2013;183(4):1113. 10.1016/j.ajpath.2013.08.002 PMC5691324 23993780

[94] The Human Protein Atlas [Internet]. Sweden: The Human Protein Atlas Project; c2003–2025 [cited 2024 Jun 2]. https://www.proteinatlas.org/.

[95] Dai S, Li L, Guo G, Peng Y, Yuan H, Li J. CCNE1 stabilizes ANLN by counteracting FZR1-mediated the ubiquitination modification to promotes triple negative breast cancer cell stemness and progression. Cell Death Discov. 2025;11(1):1–10. 10.1038/s41420-025-02518-5 PMC12064766 40346052

[96] Zhou W, Wang Z, Shen N, Pi W, Jiang W, Huang J, et al. Knockdown of ANLN by lentivirus inhibits cell growth and migration in human breast cancer. Mol Cell Biochem. 2015;398(1–2):11–9. 10.1007/s11010-014-2200-6 25223638

[97] Magnusson K, Gremel G, Rydén L, Pontén V, Uhlén M, Dimberg A, et al. ANLN is a prognostic biomarker independent of Ki-67 and essential for cell cycle progression in primary breast cancer. BMC Cancer. 2016;16(1):904. 10.1186/s12885-016-2923-8 PMC5116155 27863473

[98] Phan NN, Wang CY, Li KL, Chen CF, Chiao CC, Yu HG, et al. Distinct expression of CDCA3, CDCA5, and CDCA8 leads to shorter relapse free survival in breast cancer patient. Oncotarget. 2018;9(6):6977. 10.18632/oncotarget.24059 PMC5805530 29467944

[99] Khan B, Qahwaji R, Alfaifi MS, Athar T, Khan A, Mobashir M, et al. Deciphering molecular landscape of breast cancer progression and insights from functional genomics and therapeutic explorations followed by in vitro validation. Sci Rep. 2024;14(1):1–23. 10.1038/s41598-024-80455-6 PMC11579425 39567714

[100] Liu Y, Li J, Cao Y, Lv M. Rewired glycolysis by DTL accelerates oncometabolite L-lactate generation to promote breast cancer progression. Front Oncol. 2025;15:1583752. 10.3389/fonc.2025.1583752 PMC12086152 40391156

[101] Yang M, Huang H, Zhang Y, Wang Y, Zhao J, Lee P, et al. Identification and validation of KIF20A for predicting prognosis and treatment outcomes in patients with breast cancer. Sci Rep. 2024;14(1):31543. 10.1038/s41598-024-83362-y PMC11682246 39733078

[102] Chen X, Lu Y, Yu H, Du K, Zhang Y, Nan Y, et al. Pan-cancer analysis indicates that MYBL2 is associated with the prognosis and immunotherapy of multiple cancers as an oncogene. Cell Cycle. 2021;20(21):2291. 10.1080/15384101.2021.1982494 PMC8794527 34585645

[103] Qiu X, He H, Zeng H, Tong X, Zhang C, Liu Y, et al. Integrative transcriptome analysis identifies MYBL2 as a poor prognosis marker for osteosarcoma and a pan-cancer marker of immune infiltration. Genes Dis. 2024;11(3):101004. 10.1016/j.gendis.2023.04.035 PMC10825309 38292182

[104] Chen J, Chen X. MYBL2 is targeted by miR-143-3p and regulates breast cancer cell proliferation and apoptosis. Oncol Res. 2018;26(6):913. 10.3727/096504017X15135941182107 PMC7844795 29268817

[105] Li J, Jiang F, Wang C, Sun P, Song L, Liu J. PLCH1 overexpression promotes breast cancer progression and predicts poor prognosis through the ERK1/2-EGR1 axis. Front Oncol. 2025;15:1577114. 10.3389/fonc.2025.1577114 PMC12162997 40519297

[106] Uhrig ME, Sharma N, Maxwell P, Gomez J, Selemenakis P, Mazin AV, et al. Disparate requirements for RAD54L in replication fork reversal. Nucleic Acids Res. 2024;52(20):12390–404. 10.1093/nar/gkae828 PMC11551752 39315725

[107] Zhou Y, Qiu C, Fu Q, Li T, Zhang X, Zhu C, et al. Pan-cancer analysis of oncogenic role of RAD54L and experimental validation in hepatocellular carcinoma. J Inflamm Res. 2023;16:3997. 10.2147/JIR.S426558 PMC10503553 37719938

[108] Zhang J, Liu Y, Pu S, He J, Zhou C. Spindle and kinetochore associated complex subunit 3 accelerates breast cancer cell proliferation and invasion through the regulation of Akt/Wnt/β-catenin signaling. Breast Cancer Res Treat. 2021;186(1):247–58. 10.1007/s10549-020-06078-3 33423159

[109] Li Y, Wang W, Wu X, Ling S, Ma Y, Huang P. SLC7A5 serves as a prognostic factor of breast cancer and promotes cell proliferation through activating AKT/mTORC1 signaling pathway. Ann Transl Med. 2021;9(10):892. 10.21037/atm-21-2247 PMC8184433 34164526

[110] Tapia-Uriol P, Becerra-Goicochea L, Campos-Valderrama V, del Valle-Mendoza J, Aguilar-Luis MA, Silva-Caso WG. Characterization of intrinsic subtypes of breast cancer and their relationship with staging: an observational study. Front Med (Lausanne). 2025;12:1553910. 10.3389/fmed.2025.1553910 PMC12098373 40417678

[111] Chandler BC, Moubadder L, Ritter CL, Liu M, Cameron M, Wilder-Romans K, et al. TTK inhibition radiosensitizes basal-like breast cancer through impaired homologous recombination. J Clin Invest. 2020;130(2):958. 10.1172/JCI130435 PMC6994133 31961339

[112] Zhang S, Ding H, Deng Y, Ren Y, Zhou F, Zhang Q, et al. TTK promotes HER2+ breast cancer cell migration, apoptosis, and resistance to targeted therapy by modulating the Akt/mTOR axis. J Cancer Res Clin Oncol. 2024;150(12):512. 10.1007/s00432-024-06021-9 PMC11599621 39589549

[113] De la Rosa R, Villegas-Ruíz V, Caballero-Palacios MC, Pérez-López EI, Murata C, Zapata-Tarres M, et al. Expression of ZNF695 transcript variants in childhood B-cell acute lymphoblastic leukemia. Genes (Basel). 2019;10(9):716. 10.3390/genes10090716 PMC6771147 31527520

