# Supplementary material for: Comparative bioinformatics analysis of the Wnt pathway in breast cancer: Selection of novel biomarker panels associated with ER status
Source: Open Life Sci. 2025 Oct 8;20(1):20251173. doi: 10.1515/biol-2025-1173 (PMC12514780; doi:10.1515/biol-2025-1173)
Supplement: Supplementary Table [file biol-2025-1173-sm.pdf]

Supplementary material

Table S1: List of 183 blue module genes identified through WGCNA, served as the input for subsequent differential gene expression (DEG) analysis

Table S1: *Continued*

Blue module genes
ABCF2
ACBD7
ADSL
AEBP2
AFG3L2
AKIRIN2
ANLN
ATAD2
ATAD5
ATN1
ATP11A
B3GNT5
BAK1
BARD1
BRIX1
CAD
CALCOCO1
CCDC150
CCT3
CDC20
CDC25A
CDCA2
CDCA3
CDCA4
CDK2
CDKN2C
CDT1
CENPH
CENPO
CEP55
CHAF1A

(*Continued*)

Table S1: *Continued*

Blue module genes
CKAP2L
CKAP5
COMMD2
COPS7A
CORO1C
CYB5B
DDX47
DHFR
DHTKD1
DIAPH3
DNMT3A
DTL
EMG1
EPCAM
EXOSC2
FAM47E
FANCA
FANCC
FANCG
FBXO45
FGD4
FOXN2
GAS2L3
GCNT2
GDI2
GINS4
GNA13
GPR161
GPR180
GTSE1
HDGF
HELLS
HMGN3
HSPA14

(*Continued*)

Table S1: *Continued*

Blue module genes
IARS
ILF2
IMPA2
JRKL
KIF20A
KIF2C
KPNA2
LBR
LMNB1
LMNB2
LRP8
LRRC42
LSM2
MCM6
MED21
MEX3A
MLLT10
MPHOSPH10
MRAS
MRPL37
MRPL51
MRPS22
MSH2
MTHFD1
MTHFD1L
MTHFD2
MYBL2
NAA15
NAE1
NCAPG2
NOL11
NOP2
NSUN2
PAPD7
PATL1
PDIA6
PDK1
PFKP
PGAM5
PHB2

(*Continued*)

Table S1: *Continued*

Blue module genes
PHC1
PHF19
PIF1
PLCH1
PNO1
POLA2
POLR1B
POP1
PPP3R1
PPRC1
PRKAR1A
PSMD12
PSMD7
PUS7
RAD52
RAD54L
RANBP1
RCC2
RGMA
RHBDF2
RHEBL1
RNGTT
SASS6
SET
SKA3
SKP2
SLC25A13
SLC35F2
SLC5A6
SLC7A5
SMG5
SPAG5
SPC25
SPRYD3
SSB
SSRP1
SUOX
SUV39H2
SYNCRIP
TAF5

(*Continued*)

Table S1: *Continued*

Blue module genes
TCF7L1
TDG
TFDP1
TIMELESS
TIPRL
TK2
TMC4
TMEM201
TPCN1
TPI1
TRIM65
TTC13
TTC8
TTK
TTL
TUBA1C
TUBB
UBASH3B
UBXN2A

(*Continued*)

Table S1: *Continued*

Blue module genes
UGP2
UHRF1
UNG
UPF2
USP39
USP5
VPS39
VPS72
WDR53
WRNIP1
XPO1
XPO5
YES1
ZC3H18
ZDHHC18
ZFP91
ZNF367
ZNF384
ZNF695

Table S2: A summary of ontology of blue module genes obtained through Metascape Enrichment Analysis

GO	Category	Description	Count	%	Log10(P)	Log10(q)
R-HSA-69278	Reactome Gene Sets	Cell Cycle, Mitotic	29.00	15.85	−18.07	−13.73
GO:0000278	GO Biological Processes	mitotic cell cycle	27.00	14.75	−15.44	−11.57
GO:0010564	GO Biological Processes	regulation of cell cycle process	28.00	15.30	−13.83	−10.18
GO:0006259	GO Biological Processes	DNA metabolic process	26.00	14.21	−12.36	−8.79
WP2446	WikiPathways	Retinoblastoma gene in cancer	11.00	6.01	−11.16	−7.82
GO:0006260	GO Biological Processes	DNA replication	14.00	7.65	−10.64	−7.34
R-HSA-453279	Reactome Gene Sets	Mitotic G1 phase and G1/S transition	12.00	6.56	−9.90	−6.71
GO:0051052	GO Biological Processes	regulation of DNA metabolic process	18.00	9.84	−8.66	−5.66
R-HSA-8953854	Reactome Gene Sets	Metabolism of RNA	20.00	10.93	−7.72	−4.81
GO:0006338	GO Biological Processes	chromatin remodeling	18.00	9.84	−6.72	−3.97
GO:0034504	GO Biological Processes	protein localization to nucleus	10.00	5.46	−6.56	−3.87
GO:1901989	GO Biological Processes	positive regulation of cell cycle phase transition	8.00	4.37	−6.15	−3.57
WP241	WikiPathways	One carbon metabolism	5.00	2.73	−6.10	−3.55
M258	Canonical Pathways	PID BARD1 PATHWAY	5.00	2.73	−6.10	−3.55
WP4352	WikiPathways	Ciliary landscape	10.00	5.46	−6.07	−3.55
GO:0065004	GO Biological Processes	protein-DNA complex assembly	10.00	5.46	−5.84	−3.34
R-HSA-6790901	Reactome Gene Sets	rRNA modification in the nucleus and cytosol	6.00	3.28	−5.73	−3.24
GO:0006310	GO Biological Processes	DNA recombination	10.00	5.46	−5.55	−3.10
GO:0051983	GO Biological Processes	regulation of chromosome segregation	7.00	3.83	−4.79	−2.43
WP2363	WikiPathways	Gastric cancer network 2	4.00	2.19	−4.45	−2.17

Table S3: A summary of processes and corresponding *p*-value scores for genes within the four major networks associated with ER-linked Wnt genes, as identified through Metascape enrichment analysis

Color	MCODE	GO	Description	Log10(P)
red	MCODE_1	GO:0006260	DNA replication	−6.70
red	MCODE_1	R-HSA-1640170	Cell Cycle	−6.60
red	MCODE_1	R-HSA-8953854	Metabolism of RNA	−6.40
blue	MCODE_2	GO:0034504	protein localization to nucleus	−6.70
blue	MCODE_2	R-HSA-68886	M Phase	−5.40
blue	MCODE_2	hsa04210	Apoptosis	−5.10
green	MCODE_3	GO:0140694	non-membrane-bounded organelle assembly	−4.10
purple	MCODE_4	R-HSA-8951664	Neddylation	−6.30

Table S4: Gene components included in each signature

ER-associated multigene signature components linked to Overall Survival	ER-associated multigene signature components linked to Disease Free Survival	Multigene signature components for tumor-normal classification linked to Overall Survival	Multigene signature components for tumor-normal classification linked to Disease Free Survival
TTC8, SLC5A7, PLCH1	*ZNF695, SLC7A5, PLCH1*	*SPC25, ANLN, KPNA2, SLC7A5*	*SPC25, KIF20A, SKA3, DTL, CDCA3, ANLN, TTK, RAD54L, MYBL2, ZNF695, SLC7A5*
